# Sociodemographic and Lifestyle Factors are Associated with the Use of Dietary Supplements in a Korean Population

**DOI:** 10.2188/jea.JE20090064

**Published:** 2010-05-05

**Authors:** Jeongseon Kim, Jung-Sug Lee, Aesun Shin, Myung-Hee Kang, Dong-Soon Shin, Hae-Rang Chung, Woo-Kyoung Kim

**Affiliations:** 1 Cancer Epidemiology Branch, Division of Cancer Epidemiology and Management, Research Institute, National Cancer Center, Gyeonggi-do, Korea; 2 Department of Food and Nutrition, Han Nam University, Daejeon, Korea; 3 Department of Food and Nutrition, Kyung Nam University, Kyungnam, Korea; 4 Nutrition for the Future Inc., Seoul, Korea; 5 Department of Food and Nutrition, Dankook University, Gyeonggi-do, Korea

**Keywords:** sociodemographics, lifestyle, dietary supplements, factors

## Abstract

**Objective:**

The use of dietary supplements has been increasing rapidly in Korea over the last decade. The aim of this study was to investigate associations between the pattern of dietary supplement use and the sociodemographic/lifestyle characteristics of Korean consumers.

**Methods:**

Participants were asked to complete a self-administered questionnaire on their sociodemographic and lifestyle characteristics, perceived health status, and regular dietary supplement use.

**Results:**

A total of 697 men and 832 women completed the questionnaire. Of the respondents, 44.3% of the men and 53.2% of the women used some kind of dietary supplement regularly. Dietary supplement users were more likely to be women (*P* < 0.001), to be older than 50 years (*P* < 0.001), to have a higher household income (*P* = 0.003), to engage in moderate or vigorous physical activity (*P* < 0.032), to perceive themselves as healthy (*P* = 0.026), and to have received a diagnosis of a chronic disease (*P* < 0.001). In addition, the type of dietary supplements used varied with respect to sociodemographic and lifestyle factors. Among dietary supplement users, men preferred ginseng, and older respondents were more likely to use carbohydrate supplements and less likely to use lipid supplements. Those who had a lower BMI, were ex-smokers, or were nondrinkers preferred either vitamins or minerals. Those who were highly physical active or were nondrinkers tended to prefer either vitamin/mineral complexes or carbohydrate supplements.

**Conclusions:**

The use of dietary supplements was related to sociodemographic and lifestyle factors in a Korean population.

## INTRODUCTION

Dietary supplements are widely available for purchase both in shops and from online vendors. More than 70% of the US population uses some form of dietary supplements daily, with vitamin and mineral supplements being the most common.^1^^,^^2^ The National Health and Nutrition Examination Survey (NHANES) 1999–2000 data, which are the most recent nationally representative data on comprehensive dietary supplement use, indicated that more than half of US adults aged 20 years or older took at least 1 dietary supplement.^3^ The use of dietary supplements is increasing in Korea and in many other countries throughout the world. Sales of commercially available dietary supplements in Korea have grown tremendously over the last decade, and their variety and number continue to increase. Marketing data show a dramatic rise in dietary supplement sales in Korea, and the market was worth approximately US $25 billion in 2006.^4^ An analysis of the Korean NHANES in the year 2001 reported that 31.5% of Koreans (30.7% of men, 34.7% of women) over 20 years of age had taken dietary supplements regularly in the previous year.^5^

The use of dietary supplements is of interest to epidemiologists because supplements can be an important exposure variable or a confounding factor for many diseases and disorders. The existence of differences in sociodemographic and lifestyle characteristics between users and nonusers of dietary supplements would demonstrate the importance of including a supplement assessment in both the planning and analysis of any epidemiologic study of diet or lifestyle characteristics and health. To investigate such differences, many studies have been designed to investigate dietary supplement use with respect to demographic and lifestyle factors such as sex, age, race, economic status, obesity, smoking, and alcohol consumption. These associations have been investigated extensively in Western countries, where supplements have been widely used, but few such studies have been conducted in non-Western countries, including Korea. In the present study, we investigated the pattern of dietary supplement use in Korean consumers over 20 years of age and the associations between supplement use and several sociodemographic and lifestyle characteristics.

## METHODS

### Study participants

The participants in the present study were men and women aged 20 to 78 years who were enrolled for health check-ups at the Center for Cancer Prevention and Detection at the National Cancer Center in South Korea. Among the 1761 people who visited and provided written informed consent for study participation from October 2007 through March 2008, a total of 1529 (697 men and 832 women) who completed the questionnaire on dietary supplement use were eligible for this study (response rate: 86.8%). The study protocol was approved by the Institutional Review Board of the National Cancer Center.

### Definition of dietary supplement users

In this study, the operational definition of a dietary supplement was any pill, capsule, coated tablet, drops, powder, or beverage marketed primarily for its nutritional value or putative health-enhancing function. Users of dietary supplements were defined as those who had taken a supplement regularly, ie, at least 4 times a week during the preceding 2 years.

### Data collection and measurement

Participants were asked to complete a self-administered questionnaire on their sociodemographic characteristics, lifestyle characteristics, perceived health status, and regular dietary supplement use in the previous 2 years. Information on age, sex, region of residence, education level, and household income was collected. Age, region of residence, education level, and household income were categorized into the following groups: age (20–49, 50–64, ≥65 years); region of residence according to population size (large city, over 500 000; small city, 150 000–500 000; rural area, under 150 000); education (middle school or lower, high school, college or higher); household income (<2 000 000 won; 2 000 000–3 999 000; 4 000 000–6 999 000; ≥7 000 000). With regard to lifestyle characteristics, information was obtained about body mass index (BMI), smoking and drinking status, perceived health status, and personal medical history of chronic diseases such as cancer, cardiovascular diseases, diabetes, and bone-related diseases. BMI, smoking, drinking, perceived health status, and disease history were categorized into the following groups: BMI (<18.5 kg/m^2^, 18.5–22.9 kg/m^2^, 23–24.9 kg/m^2^, ≥25.0 kg/m^2^), smoking (current smoker, ex-smoker, nonsmoker), drinking (drinker, nondrinker), perceived health status (good, moderate, poor), history of chronic disease (yes, no). Physical activity was measured using metabolic equivalents (METs). A MET is a multiple of the resting metabolic rate and was calculated using the short form (version 2.0, April 2004) of the International Physical Activity Questionnaire. Physical activity was classified into 3 groups (low activity, <600 MET-minutes/week; moderate activity, 600–<3000 MET-minutes/week; high activity, ≥3000 MET-minutes/week). Participants were also asked whether they had taken dietary supplements regularly within the previous 2 years.

### Data analysis

The characteristics of the study population were described with numbers and percentages of participants in each category, and the chi-square test was used to compare the distributions of characteristics between dietary supplements users and nonusers. The *t* test was used to compare the mean age between dietary supplements users and nonusers. Using logistic regression methods, odds ratios (ORs) and 95% confidence intervals (CIs) were calculated to examine the effects of sociodemographic and lifestyle variables on the use of any kind of dietary supplement. All statistical analysis was performed using SAS software (version 9.12, Cary, NC, USA); the level of significance was *P* < 0.05.

## RESULTS

Table 1 shows the general characteristics of the participants. About 44.3% of the men and 53.2% of the women took dietary supplements regularly. The mean age was 51.7 years for dietary supplement users and 47.5 years for nonusers. Female sex (*P* < 0.001), age over 50 years (*P* < 0.001), residence in a large city (*P* = 0.005), high income (*P* = 0.003), high level of physical activity (*P* = 0.032), and being a nonsmoker (*P* = 0.046) were all associated with the use of dietary supplements. In addition, supplement users were more likely to perceive themselves as healthy (*P* = 0.026) and to have received a diagnosis of a chronic disease (*P* < 0.001). The crude and adjusted ORs with 95% CIs were calculated for use of each type of dietary supplement with respect to sociodemographic/lifestyle variables. Dietary supplement users were more likely to be female (OR, 1.92; 95% CI, 1.30–2.83), older (2.77, 2.06–3.70 for age 50–64 years; 4.48, 2.46–8.14 for age ≥65 years), and have a higher income (1.69, 1.07–2.68). ORs (95% CIs) for those who reported moderate and high levels of physical activity were 1.81 (1.33–2.46) and 1.55 (1.11–2.16), respectively. In addition, those who had received a diagnosis of chronic disease were significantly more likely to use dietary supplements (OR, 1.37; 95% CI, 1.00–1.88).

**Table 1.** Association between the use of dietary supplements and sociodemographic/lifestyle factors

Variable	Nonusers (%) (*n* = 777)	Users^a^ (%) (*n* = 752)	*P* value^b^	OR (95% CI)	Adjusted OR^c^ (95% CI)
Sex					
Male	388 (55.7)^d^	309 (44.3)	<0.001	1.00	1.00
Female	389 (46.8)	443 (53.2)		1.43 (1.17–1.75)	1.92 (1.30–2.83)
Age (years)^e^	47.5 ± 0.3	51.7 ± 0.3	<0.001		
Age group			<0.001		
20–49	492 (60.7)	319 (39.3)		1.00	1.00
50–64	239 (41.4)	339 (58.6)		2.19 (1.76–2.72)	2.77 (2.06–3.70)
≥65	38 (34.5)	72 (65.5)		2.92 (1.93–4.44)	4.48 (2.46–8.14)
Residential area					
Large city	179 (44.9)	220 (55.1)	0.005	1.00	1.00
Small city	490 (52.5)	443 (47.5)		0.74 (0.58–0.93)	0.88 (0.65–1.18)
Rural area	83 (59.3)	57 (40.7)		0.56 (0.38–0.83)	0.69 (0.42–1.15)
Education level					
Middle school or lower	110 (50.5)	108 (49.5)	0.612	1.00	1.00
High school	277 (52.5)	251 (47.5)		0.92 (0.67–1.27)	1.03 (0.66–1.62)
College or higher	378 (49.7)	383 (50.3)		1.03 (0.76–1.40)	1.25 (0.80–1.95)
Monthly income (×1000 won)					
<2000	100 (49.0)	104 (51.0)	0.003	1.00	1.00
2000–3999	245 (56.2)	191 (43.8)		0.75 (0.54–1.05)	0.85 (0.56–1.29)
4000–6999	194 (51.2)	185 (48.8)		0.92 (0.65–1.29)	1.25 (0.81–1.95)
≥7000	142 (42.7)	190 (57.2)		1.29 (0.91–1.83)	1.69 (1.07–2.68)
Body mass index (kg/m^2^)					
<18.5	21 (55.3)	17 (44.7)	0.132	0.76 (0.40–1.48)	0.79 (0.32–1.96)
18.5–22.9	292 (48.6)	309 (51.4)		1.00	1.00
23–24.9	204 (48.8)	214 (51.2)		0.99 (0.77–1.27)	0.86 (0.62–1.19)
≥25	260 (55.1)	212 (44.9)		0.77 (0.61–0.98)	0.67 (0.48–0.92)
Physical activity (METs^f^)					
Low	246 (54.6)	205 (45.4)	0.032	1.00	1.00
Moderate	230 (46.9)	260 (53.1)		1.36 (1.05–1.75)	1.81 (1.33–2.46)
High	185 (46.9)	209 (53.1)		1.36 (1.03–1.78)	1.55 (1.11–2.16)
Cigarette smoking					
Current smoker	164 (57.9)	119 (42.1)	0.046	1.00	1.00
Ex-smoker	183 (50.0)	183 (50.0)		1.38 (1.01–1.88)	1.28 (0.87–1.89)
Nonsmoker	413 (49.6)	419 (49.7)		1.40 (1.07–1.84)	0.83 (0.53–1.31)
Alcohol drinking					
Drinker	459 (52.9)	409 (47.1)	0.113	1.00	1.00
Nondrinker	297 (48.7)	313 (51.3)		1.18 (0.96–1.46)	0.87 (0.64–1.17)
Perceived health status					
Good	218 (48.3)	233 (51.7)	0.026	1.00	1.00
Moderate	381 (53.6)	330 (46.4)		0.81 (0.64–1.03)	0.89 (0.67–1.20)
Poor	145 (45.0)	177 (55.0)		1.14 (0.86–1.52)	1.15 (0.79–1.68)
Diagnosis of chronic disease^g^					
No	231 (61.4)	145 (38.6)	<0.001	1.00	1.00
Yes	540 (47.3)	601 (52.7)		1.77 (1.40–2.25)	1.37 (1.00–1.88)

^a^A user of dietary supplements was defined as a person who takes them regularly, ie, at least 4 times a week during the preceding 2 years.

^b^The *t* test was used to compare mean age between dietary supplement users and nonusers; the chi-square test was used to compare other general characteristics.

OR, Odds ratio; CI, Confidence interval.

^c^Odds ratios adjusted for all other significant variables in the model.

^d^*n* (%).

^e^Mean ± standard error.

^f^A MET (metabolic equivalent) is a multiple of the resting metabolic rate and was calculated using the short form (version 2.0, April 2004) of the International Physical Activity Questionnaire (low activity: <600 MET-minutes/week, moderate activity: 600–<3000 MET-minutes/week, high activity: ≤3000 MET-minutes/week).

^g^Chronic diseases such as cancer, cardiovascular diseases, diabetes, and bone-related diseases.

Participants consumed the following types of dietary supplements: vitamins, minerals, vitamin/mineral complexes, ginseng, carbohydrate-based supplements (including glucosamine, chitosan, chitooligosaccharide, aloe, and dietary fiber), lipid-based supplements (including γ-linolenic acid, eicosapentaenoic acid/docosahexaenoic acid (EPA/DHA), ω-3 fatty acid, squalene, and other lipids), seaweeds (including spirulina and chlorella), and others, such as royal jelly, soybean protein, amino acids, yeast, lactic acid bacteria, mushrooms, green tea extract, and other plant extracts (Table 2). There were a number of associations between sociodemographic/lifestyle variables and the type of dietary supplements used. Women were less likely to consume ginseng (OR, 0.55; 95% CI, 0.35–0.84), and older respondents tended to consume more carbohydrates (2.90, 1.88–4.48 for age 50–64 years; 4.28, 2.31–7.93 for age ≥65 years) and fewer lipids (0.65, 0.46–0.91 for age 50–64 years; 0.53, 0.29–0.99 for age ≥65 years). In addition, vitamin supplement use was positively associated with having a BMI lower than 18.5 (2.76, 1.01–7.52) and being an ex-smoker (2.09, 1.18–3.69). In particular, a high level of physical activity was associated with a decreased tendency to consume vitamin/mineral complexes (0.56, 0.33–0.96). The use of mineral supplements was significantly higher among nondrinkers (1.59, 1.03–2.43), whereas the use of carbohydrate supplements was much lower (0.59, 0.39–0.90). The mean number of dietary supplement types consumed among supplement users was 1.8 for men and 2.4 for women (data not shown). The percentage of users who took more than 1 supplement type, ie, multiple users (defined as the number of users of each supplement divided by the total number of users × 100), was 56.1% (45.9% of men, 63.2% of women). Women were more likely to take more than 1 type of dietary supplement than were men (1.96, 1.10–3.51). Other sociodemographic and lifestyle factors were not associated with use of more than 1 supplement.

**Table 2.** Association between the use of various types of dietary supplements and sociodemographic/lifestyle characteristics

Characteristic	Vitamins		Minerals		Vitamin/mineral complexes		Ginseng		Carbohydrates^a^		Lipids^b^		Seaweeds^c^		Others^d^		Multiple types	
								
	*n* (%)^f^	OR (95% CI)^g^	*n* (%)	OR (95% CI)	*n* (%)	OR (95% CI)	*n* (%)	OR (95% CI)	*n* (%)	OR (95% CI)	*n* (%)	OR (95% CI)	*n* (%)	OR (95% CI)	*n* (%)	OR (95% CI)	*n* (%)	OR (95% CI)
Sex																		
Male	100 (32.4)	1.00	41 (13.3)	1.00	56 (18.1)	1.00	161 (52.1)	1.00	65 (21.0)	1.00	101 (32.7)	1.00	27 (8.7)	1.00	18 (5.8)	1.00	142 (45.9)	1.00
Female	127 (28.7)	0.58 (0.35–0.96)	170 (38.4)	1.72 (0.93–3.18)	94 (21.2)	1.08 (0.60–1.94)	169 (38.1)	0.55 (0.35–0.84)	189 (42.7)	1.68 (0.97–2.92)	222 (50.1)	1.54 (0.96–2.47)	73 (16.5)	1.95 (0.88–4.30)	33 (7.4)	1.28 (0.49–3.35)	280 (63.2)	1.96 (1.10–3.51)
Age (years)																		
20–49	85 (26.6)	1.00	76 (23.8)	1.00	66 (20.7)	1.00	147 (46.1)	1.00	62 (19.4)	1.00	136 (42.6)	1.00	51 (16.0)	1.00	24 (7.5)	1.00	159 (49.8)	1.00
50–64	107 (31.6)	1.08 (0.74–1.57)	103 (30.4)	1.12 (0.73–1.70)	61 (18.0)	0.77 (0.50–1.20)	147 (43.4)	0.96 (0.69–1.33)	143 (42.2)	2.90 (1.88–4.48)	145 (42.8)	0.65 (0.46–0.91)	37 (10.9)	0.53 (0.30–0.96)	24 (7.1)	1.03 (0.51–2.10)	203 (59.9)	1.45 (0.98–2.16)
≥65	28 (38.9)	0.90 (0.46–1.73)	24 (33.3)	0.75 (0.35–1.59)	20 (27.8)	1.26 (0.63–2.50)	28 (38.9)	0.72 (0.40–1.30)	38 (52.8)	4.28 (2.31–7.93)	30 (41.7)	0.53 (0.29–0.99)	9 (12.5)	1.17 (0.47–2.94)	3 (4.2)	0.84 (0.23–3.16)	46 (63.9)	1.53 (0.74–3.17)
Residential area																		
Large city	75 (34.1)	1.00	55 (25.0)	1.00	48 (21.8)	1.00	105 (47.7)	1.00	68 (30.9)	1.00	82 (37.3)	1.00	27 (12.3)	1.00	16 (7.3)	1.00	126 (57.3)	1.00
Small city	127 (28.7)	0.77 (0.53–1.11)	133 (30.0)	1.06 (0.69–1.62)	94 (21.2)	0.83 (0.55–1.27)	184 (41.5)	0.86 (0.61–1.18)	150 (33.9)	0.95 (0.64–1.41)	212 (47.9)	1.65 (1.15–2.36)	64 (14.4)	0.98 (0.56–1.71)	29 (6.5)	0.91 (0.44–1.88)	253 (62.2)	0.99 (0.66–1.48)
Rural area	16 (28.1)	0.76 (0.39–1.49)	17 (29.8)	1.15 (0.54–2.46)	5 (8.8)	0.51 (0.19–1.35)	32 (56.1)	1.23 (0.69–2.17)	20 (35.1)	1.02 (0.51–2.06)	14 (24.6)	0.92 (0.44–1.91)	6 (10.5)	1.23 (0.44–3.44)	5 (8.8)	2.03 (0.68–6.05)	28 (49.1)	0.74 (0.36–1.54)
Education level																		
Middle school ​ ​ or lower	19 (17.6)	1.00	36 (33.3)	1.00	11 (10.2)	1.00	42 (38.9)	1.00	45 (41.7)	1.00	36 (33.3)	1.00	7 (6.5)	1.00	6 (5.6)	1.00	58 (53.7)	1.00
High school	68 (27.1)	1.89 (0.93–3.82)	80 (31.9)	0.77 (0.43–1.37)	38 (15.1)	1.36 (0.60–3.13)	121 (48.2)	1.12 (0.68–1.85)	83 (33.1)	0.71 (0.41–1.24)	114 (45.4)	1.49 (0.82–2.70)	32 (12.7)	0.90 (0.36–2.28)	12 (4.8)	0.74 (0.21–2.56)	138 (55.0)	0.68 (0.37–1.27)
College or higher	137 (35.8)	2.29 (1.15–4.54)	93 (24.3)	0.72 (0.40–1.30)	99 (25.8)	2.17 (0.98–4.78)	161 (42.0)	0.63 (0.39–1.04)	124 (32.4)	0.81 (0.47–1.39)	170 (44.4)	1.62 (0.90–2.91)	59 (15.4)	1.07 (0.44–2.62)	32 (8.4)	1.41 (0.45–4.37)	222 (58.0)	1.19 (0.64–2.23)
Monthly income (×1000 won)																		
<2000	28 (26.9)	1.00	42 (40.4)	1.00	23 (22.1)	1.00	45 (43.3)	1.00	43 (41.3)	1.00	41 (39.4)	1.00	11 (10.6)	1.00	5 (4.8)	1.00	60 (57.7)	1.00
2000–3999	67 (35.1)	0.86 (0.49–1.50)	51 (26.7)	0.86 (0.48–1.52)	31 (16.2)	0.61 (0.31–1.19)	75 (39.3)	0.91 (0.54–1.52)	63 (33.3)	1.02 (0.59–1.76)	81 (42.4)	1.31 (0.77–2.22)	26 (13.6)	2.39 (0.86–6.66)	12 (6.3)	1.04 (0.34–3.17)	99 (51.8)	0.83 (0.45–1.51)
4000–6999	55 (29.7)	0.58 (0.32–1.05)	50 (27.0)	1.09 (0.60–1.96)	38 (20.5)	0.80 (0.41–1.56)	69 (37.3)	0.97 (0.57–1.64)	68 (36.8)	1.27 (0.72–2.26)	82 (44.3)	1.42 (0.83–2.45)	17 (9.2)	1.37 (0.46–4.09)	11 (5.9)	0.76 (0.23–2.53)	100 (54.0)	0.85 (0.45–1.61)
≥7000	50 (26.3)	0.51 (0.28–0.92)	32 (16.8)	0.76 (0.41–1.40)	44 (23.2)	0.85 (0.44–1.63)	111 (58.4)	1.43 (0.86–2.39)	46 (24.2)	0.89 (0.49–1.60)	67 (35.3)	1.03 (0.59–1.80)	30 (15.8)	2.69 (0.95–7.63)	16 (8.4)	1.22 (0.39–3.77)	107 (56.3)	1.18 (0.62–2.23)
BMI (kg/m^2^)																		
<18.5	6 (35.3)	2.76 (1.01–7.52)	3 (17.6)	0.35 (0.05–2.70)	2 (11.8)	0.70 (0.16–3.13)	8 (47.1)	1.00 (0.32–3.11)	4 (23.5)	1.05 (0.23–4.73)	7 (41.2)	0.34 (0.08–1.50)	4 (23.5)	1.63 (0.44–6.04)	3 (17.6)	2.32 (0.48–11.22)	10 (58.5)	0.82 (0.22–3.11)
18.5–22.9	89 (28.8)	1.00	92 (29.8)	1.00	63 (20.4)	1.00	136 (44.0)	1.00	86 (27.8)	1.00	141 (45.6)	1.00	50 (16.2)	1.00	18 (5.8)	1.00	173 (56.0)	1.00
23–24.9	61 (28.5)	0.89 (0.57–1.38)	63 (29.4)	1.68 (1.08–2.62)	45 (21.0)	0.92 (0.57–1.50)	97 (45.3)	0.90 (0.62–1.29)	86 (40.2)	1.27 (0.81–2.00)	89 (41.6)	0.99 (0.67–1.46)	18 (8.4)	0.48 (0.23–1.00)	19 (8.9)	1.21 (0.55–2.65)	125 (58.4)	1.23 (0.78–1.95)
≥25	71 (33.5)	1.09 (0.70–1.70)	53 (25.0)	0.95 (0.57–1.59)	40 (18.9)	0.90 (0.54–1.52)	89 (42.0)	0.78 (0.53–1.14)	78 (36.8)	1.45 (0.91–2.29)	86 (40.6)	1.14 (0.76–1.70)	28 (13.2)	1.11 (0.59–2.09)	11 (5.2)	0.77 (0.31–1.95)	114 (53.8)	1.32 (0.82–2.11)
Physical activity (METs^e^)																		
Low	57 (27.8)	1.00	57 (27.8)	1.00	50 (24.4)	1.00	83 (40.5)	1.00	76 (37.1)	1.00	100 (48.8)	1.00	26 (12.7)	1.00	15 (7.3)	1.00	111 (54.2)	1.00
Moderate	83 (31.9)	1.37 (0.89–2.13)	55 (21.2)	0.90 (0.57–1.43)	59 (22.7)	0.93 (0.59–1.46)	115 (44.2)	1.12 (0.77–1.61)	85 (32.7)	0.90 (0.59–1.37)	100 (38.5)	1.00 (0.69–1.44)	33 (12.7)	1.16 (0.61–2.22)	17 (6.5)	0.84 (0.39–1.81)	145 (55.8)	1.39 (0.90–2.15)
High	69 (33.0)	1.42 (0.89–2.27)	68 (32.5)	1.23 (0.76–1.97)	29 (13.9)	0.56 (0.33–0.96)	105 (50.2)	1.23 (0.83–1.80)	65 (31.1)	0.88 (0.55–1.39)	84 (40.2)	0.83 (0.55–1.24)	33 (15.8)	1.58 (0.82–3.04)	15 (7.2)	0.84 (0.36–1.93)	121 (57.9)	1.44 (0.89–2.31)
Smoking																		
Current smoker	27 (22.7)	1.00	15 (12.6)	1.00	24 (20.2)	1.00	63 (52.9)	1.00	24 (20.2)	1.00	46 (38.7)	1.00	10 (8.4)	1.00	8 (6.7)	1.00	50 (42.0)	1.00
Ex-smoker	72 (39.3)	2.09 (1.18–3.69)	36 (19.7)	2.29 (0.99–5.34)	34 (18.6)	0.79 (0.43–1.45)	88 (48.1)	0.80 (0.51–1.26)	40 (21.9)	1.06 (0.54–2.08)	64 (35.0)	0.76 (0.45–1.27)	21 (11.5)	1.10 (0.45–2.71)	11 (6.0)	0.84 (0.29–2.42)	95 (51.9)	1.58 (0.90–2.77)
Nonsmoker	120 (28.6)	1.25 (0.65–2.38)	151 (36.0)	1.58 (0.67–3.73)	87 (20.8)	0.68 (0.34–1.37)	168 (40.1)	0.92 (0.55–1.53)	171 (40.8)	1.76 (0.89–3.51)	199 (47.5)	0.82 (0.47–1.42)	62 (14.8)	0.77 (0.30–2.01)	32 (7.6)	1.00 (0.32–3.15)	259 (61.8)	1.49 (0.79–2.81)
Drinking																		
Drinker	121 (29.6)	1.00	84 (20.5)	1.00	73 (17.8)	1.00	201 (49.1)	1.00	118 (28.9)	1.00	154 (37.7)	1.00	46 (11.2)	1.00	29 (7.1)	1.00	209 (51.1)	1.00
Nondrinker	97 (31.0)	1.06 (0.71–1.58)	113 (36.1)	1.59 (1.03–2.43)	73 (23.3)	1.24 (0.78–1.95)	120 (38.3)	0.89 (0.63–1.25)	115 (36.7)	0.59 (0.39–0.89)	155 (49.5)	1.03 (0.73–1.47)	49 (15.7)	1.10 (0.63–1.95)	21 (6.7)	0.61 (0.29–1.31)	193 (61.7)	1.09 (0.72–1.67)
Perceived health status																		
Good	85 (36.5)	1.00	57 (24.5)	1.00	47 (20.2)	1.00	98 (42.1)	1.00	77 (33.0)	1.00	104 (44.6)	1.00	28 (12.0)	1.00	18 (7.7)	1.00	134 (57.5)	1.00
Moderate	102 (30.9)	0.83 (0.56–1.21)	99 (30.0)	1.09 (0.70–1.72)	63 (19.1)	0.92 (0.58–1.45)	155 (47.0)	1.30 (0.93–1.82)	112 (33.9)	1.01 (0.67–1.52)	134 (40.6)	0.90 (0.63–1.28)	44 (13.3)	1.35 (0.73–2.47)	18 (5.5)	0.83 (0.38–1.80)	183 (55.4)	0.91 (0.60–1.38)
Poor	36 (20.3)	0.65 (0.40–1.07)	52 (29.4)	1.16 (0.69–1.96)	39 (22.0)	1.10 (0.64–1.90)	71 (40.1)	0.94 (0.62–1.45)	62 (35.0)	1.23 (0.74–2.04)	82 (46.3)	0.84 (0.54–1.30)	26 (14.7)	1.33 (0.64–2.74)	14 (7.9)	1.36 (0.58–3.22)	99 (55.9)	0.93 (0.55–1.56)
Diagnosis of chronic disease																		
No	34 (23.4)	1.00	40 (27.6)	1.00	22 (15.2)	1.00	53 (36.6)	1.00	51 (35.2)	1.00	65 (44.8)	1.00	19 (13.1)	1.00	7 (4.8)	1.00	79 (54.5)	1.00
Yes	192 (31.9)	1.08 (0.67–1.73)	171 (28.5)	0.90 (0.55–1.48)	127 (21.1)	1.10 (0.64–1.89)	274 (45.6)	1.33 (0.87–2.03)	201 (33.4)	0.57 (0.36–0.91)	258 (42.9)	0.88 (0.59–1.31)	79 (13.1)	0.86 (0.45–1.66)	44 (7.3)	1.40 (0.53–3.73)	341 (56.7)	1.01 (0.63–1.61)

^a^Including glucosamine, chitosan, chitooligosaccharide, aloe, and dietary fiber.

^b^Including gamma linolenic acid, EPA/DHA, ω-3 fatty acid, squalene, and other lipids.

^c^Including spirulina and chlorella.

^d^Including royal jelly, soybean protein, amino acids, yeast, lactic acid bacteria, mushrooms, green tea extract, and other plants extract.

^e^A MET is multiple of resting metabolic rate and was calculated using the short form (version 2.0, April 2004) of the International Physical Activity Questionnaire (low activity: <600 MET-minutes/week, moderate activity: 600–<3000 MET-minutes/week, high activity: ≤3000 MET-minutes/week).

OR, Odds ratio; CI, Confidence interval.

^f^*n*, Number of users of a supplement type; %, Percentage of users (number of users of a supplement type/total number of supplement users × 100).

^g^Odds ratio adjusted for all other significant variables in the model.

## DISCUSSION

In this study, the prevalence of dietary supplement use and its association with sociodemographic and lifestyle characteristics were investigated. Sex, age, area of residence, monthly income, BMI, physical activity, smoking, perceived health status, and a diagnosis of chronic disease were associated with dietary supplement use. In particular, being female, being older, earning a higher income, and having a high level of physical activity were strongly associated with the use of dietary supplements. As compared with participants with a normal BMI, those with lower and higher BMIs had a tendency not to use dietary supplements. The current study showed that participants with chronic diseases tended to use dietary supplements more than those without such conditions, probably because greater awareness of their health status led them to seek treatments for these conditions. Among supplement users, men preferred ginseng, and older respondents preferred carbohydrate supplements and were less likely to consume lipid supplements. Those who had lower BMIs, were ex-smokers, or were nondrinkers preferred vitamin or mineral supplements. Those who had a high level of physical activity or were nondrinkers tended to prefer vitamin/mineral complexes or carbohydrate supplements.

The disparities in the findings of studies on dietary supplement use might in part be attributable to differences in the way supplement use was defined.^6^ For example, in 1 study, supplement users were defined as people who had taken a supplement on the day of the study^7^; whereas, in another, they were defined as those who had used supplements in the past 2 weeks.^8^ In a cohort study of French women, dietary supplement users were defined as those who took supplements at least 3 times a week.^9^ In the present study, users of dietary supplements were defined as those who had taken them at least 4 times a week during the previous 2 years. Using this definition, about half of the study participants used dietary supplements regularly. This stricter definition, with its higher frequency requirement, may be 1 reason why the prevalence of dietary supplement use was lower in this study than in others. However, the figure in the current study is higher than that reported in the 2005 Korean NHANES,^10^ which reported the prevalence rate for the use of dietary supplements among adults in Korea. The main reason for the higher rate in the present report is that it was designed to enroll individuals visiting a hospital for cancer screening, which is a population of higher socioeconomic status. In a survey of older adults in the United States conducted by Marinac et al,^11^ 21% of respondents reported currently taking at least 1 herbal product or dietary supplement. Another study of adults aged 50 to 59 years in France and Northern Ireland found that 15% of French and 21% of Northern Irish participants used vitamin supplements.^12^

Several studies have examined the association of age with dietary supplement use. The NHANES 2001–2002 and NHANES III 1988–1994 surveys in the United States found that users of multiple supplements were primarily older.^13^ In a study of non-vitamin, non-mineral (NVNM) dietary supplements in NHANES III, the data suggested associations between NVNM supplement use and age.^14^ Also, in data from the California Teachers cohort study, users of multivitamins or other specific supplements tended to be older.^15^ In data from a population of French women, supplement users were more likely to be older than nonusers.^9^ In addition, in a Japanese study the prevalence of supplement use was higher among the elderly.^16^ In contrast, in a longitudinal survival study of NVNM use in an aging population, younger age was directly related to NVNM use.^17^

Regarding sex differences in the use of dietary supplements, among older adults in the United States, women—especially white women with at least a college education—were more likely to take dietary supplements.^11^ Glucosamine, garlic, *Echinacea*, and *Gingko biloba* were the most frequently cited substances used by those survey participants. Also, in a diverse and elderly US population, women took more supplements than did men.^18^ More women than men took calcium, likely because of the sex-specific indications for these supplements, while more men than women took lycopene and saw palmetto. Data from NHANES 2001–2002 and NHANES III 1988–1994 showed that most women consumed γ-linolenic acid and a probiotic supplement, whereas men consumed zinc, garlic, saw palmetto, and soy protein supplements.^13^

Health-related factors, such as perception of health and healthy lifestyle, have been related to dietary supplement use. The NHANES 2001–2002 and NHANES III 1988–1994 surveys reported that supplement users were more likely to describe their health as “very good” or “excellent”.^13^ The study of non-vitamin, non-mineral (NVNM) dietary supplements in the NHANES III showed that NVNM supplement use was associated with more healthful lifestyles, and that garlic and lecithin were the most frequently used supplements.^14^ In a study conducted in France and Northern Ireland, vitamin supplement use was associated with a healthier lifestyle.^12^ In another study of French women, users of supplements were more likely than nonusers to engage in leisure physical activity.^9^ A similar result was noted in a Japanese study: the prevalence of supplement users was higher among more physically active participants.^16^ Higher physical activity, which is more frequent in dietary supplement users, has been linked to leanness and lower BMI in several studies.^9^^,^^15^^,^^16^ Also, in a longitudinal survival study of NVNM use in an aging population, a history of disease was inversely related to NVNM supplement use.^17^ After controlling for confounding factors, users of NVNM supplements had lower mortality than did nonusers. Users of dietary supplements also tended to have a healthier diet than nonusers, ie, they drank less alcohol and ate more healthful food (eg, vegetables, fruit, dairy products, and fish). Among health-related behaviors, smoking status was a related factor: supplement users were less likely to be current smokers.^9^^,^^15^^–^^17^ In addition, in data from a population of French women, women who took supplements were more likely than nonusers to have had a family history of breast cancer. They were also more likely to have had regular health check-ups such as mammography, colonoscopy, Pap smear, bone densitometry testing, and cholesterol or blood pressure testing.^9^

Socioeconomic status is related to the use of dietary supplements. The US NHANES 2001–2002 and NHANES III 1988–1994 surveys showed that supplement users were more affluent.^13^ In a Japanese study, supplement users were likely to be self-employed.^16^ As for factors other than the sociodemographic factors mentioned above, area of residence, race, marital status, family structure, and education were also significantly related to supplement use. The data on French women showed that supplement users were more likely to live alone, to have few or no children, to live in the Mediterranean region, and to live in larger cities.^9^ Education has been investigated in 2 studies^9^^,^^13^: dietary supplement use was related to higher educational attainment.

There were limitations to the present study. The supplement use patterns of adults mostly residing in the Seoul metropolitan area may not reflect those in other areas of the country. Another important limitation of the study is that the data are cross-sectional; therefore, the reported associations, particularly with respect to health outcomes, cannot establish causality. Although the prevalence of dietary supplement use cannot be generalized to the national level, the internal validity of the observed association is not likely to be seriously affected by population selection. To our knowledge, the present study is the first to investigate supplement use and its associations with sociodemographic, lifestyle, and health factors in Korea. We recommend further investigation of the brand names, frequency, and duration of the dietary supplements used. The results of this study indicate that about half of the study participants took dietary supplements regularly. To accurately estimate nutrient status and potential health risks for Korean adults, it will be necessary to include nutrient intakes from food, as well as from dietary supplements. In conclusion, our study suggests that use of dietary supplement is affected by some sociodemographic and lifestyle factors.
